# Tumor cell density regulates matrix metalloproteinases for enhanced migration

**DOI:** 10.18632/oncotarget.25863

**Published:** 2018-08-24

**Authors:** Hasini Jayatilaka, Fatima G. Umanzor, Vishwesh Shah, Tomer Meirson, Gabriella Russo, Bartholomew Starich, Pranay Tyle, Jerry S.H Lee, Shyam Khatau, Hava Gil-Henn, Denis Wirtz

**Affiliations:** ^1^ Department of Chemical and Biomolecular Engineering, The Johns Hopkins University, Baltimore, MD, USA; ^2^ Johns Hopkins Physical Sciences-Oncology Center, The Johns Hopkins University, Baltimore, MD, USA; ^3^ Department of Pediatrics, Bass Center for Childhood Cancer, Stanford University, Stanford, CA, USA; ^4^ Center for Strategic Scientific Initiatives, National Cancer Institute, Bethesda, MD, USA; ^5^ Lawrence J. Ellison Institute for Transformative Medicine, University of Southern California, Los Angeles, CA, USA; ^6^ Department of Medicine/Oncology, Keck School of Medicine, University of Southern California, Los Angeles, CA, USA; ^7^ The Azrieli Faculty of Medicine, Bar-Ilan University, Safed, Israel; ^8^ Department of Oncology and Department of Pathology, Johns Hopkins University School of Medicine, Baltimore, MD, USA

**Keywords:** cell migration, matrix metalloproteinases, interleukin 6, interleukin 8

## Abstract

Matrix metalloproteinases (MMPs) may play a critical role in metastatic cancers, yet multiple human clinical trials targeting MMPs have surprisingly failed. Cancer cell density changes dramatically during the early growth of a primary tumor and during the early seeding steps of secondary tumors and has been implicated in playing an important role in regulating metastasis and drug resistance. This study reveals that the expression of MMPs is tightly regulated by local tumor cell density through the synergistic signaling mechanism of Interleukin 6 (IL-6) and Interleukin 8 (IL-8) via the JAK2/STAT3 complex. Local tumor cell density also plays a role in the responsiveness of cells to matrix metalloproteinases inhibitors (MMPI), such as Batimastat, Marimastat, Bryostatin I, and Cipemastat, where different migratory phenotypes are observed in low and high cell density conditions. Cell density-dependent MMP regulation can be directly targeted by the simultaneous inhibition of IL-6 and IL-8 receptors via Tocilizumab and Reparixin to significantly decrease the expression of MMPs in mouse xenograft models and decrease effective metastasis. This study reveals a new strategy to decrease MMP expression through pharmacological intervention of the cognate receptors of IL-6 and IL-8 to decrease metastatic capacity of tumor cells.

## INTRODUCTION

Matrix metalloproteinases (MMPs) form a family of metzincin proteases that contribute to the degradation of extracellular-matrix proteins and remodeling of the basement membrane [1–3]. Overexpression and activation of MMPs leads to the degradation of the basement membrane facilitating tumor cell invasion. MMPs may play a central role in tumor metastasis [4], a process during which cancer cells break away from the primary malignant tissue and travel through the lymphatic and vascular systems to distal sites, promoting the spread of tumorigenic cells. Following many successful pre-clinical studies using matrix metalloproteinases inhibitors (MMPIs) as anti-cancer agents, these inhibitors were tested in multiple human clinical trials [5]. Surprisingly, these clinical trials of over 50 MMPIs failed. While the failure of these clinical trials may be attributed to poor design, limited specificity, inadequate clinical end points, and poor pharmacokinetics (poor oral bioavailability and dose-limiting toxicities), a limited fundamental understanding of the specific regulators of MMPs could be an important underlying cause [6].

Tumor cell density has been shown to play an important role in regulating metastasis and drug resistance [7–9]. Cancer cell density increases as cells proliferate in a space confined by the basement membrane and the surrounding stromal matrix. These cell density-dependent phenotypes are driven by the activation of the transcription factor signal transducer and activator of transcription 3 (STAT3). STAT3 has also been shown to regulate the production of MMPs. For instance, the expression of MMP 10 is regulated through the JAK2/STAT3 signaling in adenocarcinomas [10–13]. Cell density-dependent migration has also been shown to be promoted by the signaling of Interleukin 6 (IL-6) and Interleukin 8 (IL-8) via the JAK2/STAT3 signaling [8]. Individually, these cytokines regulate the expression of MMPs in various types of cancers. IL-6 upregulates the expression of MMPs (1, 2, and 9) [14, 15], thereby increasing cell migration and tumor metastasis; while IL-8 has been implicated in the upregulation of MMP 2 [13] and mediation of MMP-9 release [16], which correlates with high-grade and invasive tumors [17]. Together, these cytokines promote tumor self-seeding by circulating cancer cells through the increased expression of MMP 1 [18]. Considering the individual influence of IL-6 and IL-8 on the regulation of MMP expression and the role STAT3 plays in driving metastasis and drug resistance, we speculated that different subgroups of MMPs - such as collagenase, gelatinase, matrilysin, stromelysin, and membrane-type - could be regulated by tumor cell density through the synergistic signaling of IL-6 and IL-8.

In this study, we show that local tumor cell density in 3D collagen I matrices regulates the expression and activity of MMPs through the synergistic paracrine signaling mechanism between IL-6 and IL-8 via the JAK2/STAT3 pathway. As tumor cells proliferate and local cell density increases, the mRNA expression of MMPs (1, 2, 3, 7, 9, 10, 11, and 14) also increases. We demonstrate that the protein expression of MMP 1, MMP 2, MMP 3, and MMP 9 are also upregulated in high cell density conditions. Further, the activity of MMP 1 and MMP 9 increased with an increase cell density. Strikingly, shRNA-mediated knockdowns of particular MMPs induces different migratory phenotypes at low and high cell densities. These different migratory phenotypes can also be observed when fibrosacroma and carcinoma cells are treated with MMPIs, including Batimastat, Marimastat, Bryostatin-1, and Cipemastat. These inhibitors have little-to-no effect on cell migration at a low cell density, but do significantly decrease cell speed at a high density, suggesting an influence of cell density on the responsiveness of cells to MMPIs. Based on these results, we tested a novel strategy to affect MMP expression through the targeting of the cell density-dependent mechanism via the cognate receptors of IL-6 (IL-6R) and IL-8 (IL-8R) using Tocilizumab and Reparixin (T+R). In two independent mouse xenograft models utilizing the MDA-MB-231 cells and patient-derived triple negative breast cancer cells, dual inhibition of IL-6R and IL-8R significantly decreased the expression of MMPs and decreased effective metastasis to the lungs and liver. Transcriptome analysis of the PDX tumors also demonstrates that overall MMP expression is decreased in the group treated with the T+R combination. These findings provide new fundamental insights into the expression of MMPs and suggest a novel strategy to reduce the expression and activity of MMPs, decrease the metastatic capacity of tumor cells.

## RESULTS

### Cell density regulates MMPs through the synergistic paracrine signaling pathway of IL-6 and IL-8

**Figure 1:**
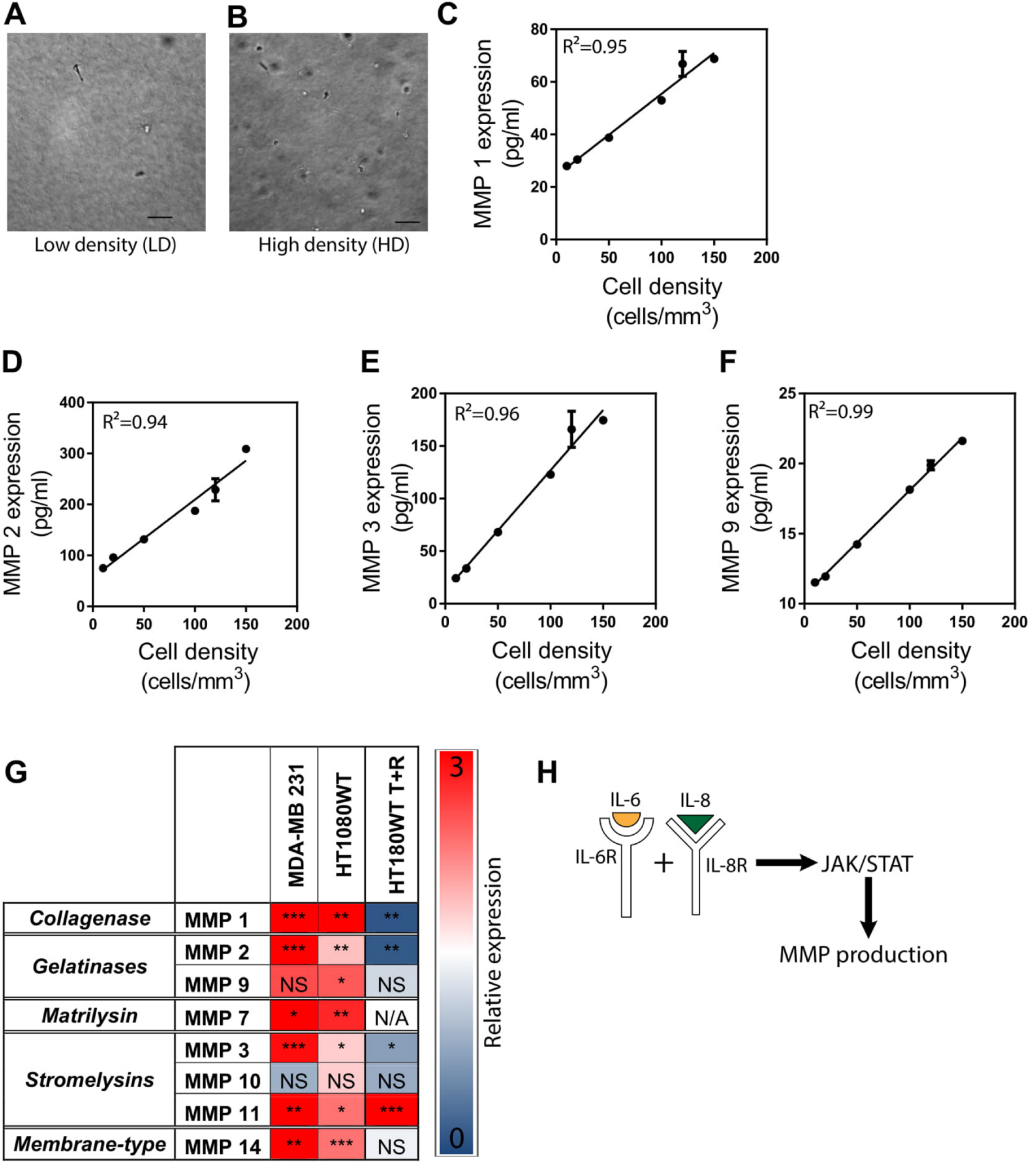
MMP expression is regulated by cancer cell density via the signaling of IL-6 and IL-8 (**A** and **B**) Phase contrast micrographs of cells at low and high density illustrating difference between cell environment. (**C**–**F**) Protein expression of MMP 1, 2, 3, and 9 linearly increase with increasing cell density. (**G**) mRNA expression of MMPs in high cell density environments relative to low cell density environments in matrix-embedded breast carcinoma cells MDA-MB-231, fibrosarcoma HT1080 cells, and fibrosarcoma HT1080 cells treated with a combination of tocilizumab and reparixin. Figure shows a decrease in relative MMP expression for HT1080 cells treated with tocilizumab and reparixin. (**H**) Illustration depicts how the synergistic signaling of interleukin 6 (IL-6) and interleukin 8 (IL-8) regulates the production of MMPs through the JAK/STAT signaling pathway. In all panels, data is represented as mean ± s.e.m. ^*^P < 0.05; ^**^P < 0.01; ^***^P < 0.001(ANOVA).

To assess the potential effect of tumor cell density on the expression of MMPs *in vitro*, we performed a Luminex multiplex assay with conditioned medium obtained from human fibrosarcoma HT1080 cells [19–22] embedded in a 3D collagen I matrix at low and high cell densities ranging from 10 cells.mm^−3^ to 150 cells.mm^−3^. (Figure 1A and 1B) We found that the protein expression of MMP 1, MMP 2, MMP 3, and MMP 9 linearly increased with increasing cell density, suggesting a cell-density-dependent expression (Figure 1C–1F). We also examined the activity of MMP 1 and MMP 9 and saw an increase in activity with increasing cell density (Supplementary Figure 1A and 1B). We further conducted PCR experiments with matrix-embedded breast carcinoma cells MDA-MB-231 and fibrosarcoma HT1080 cells at low density (LD), 10 cells.mm^−3^, and high density (HD), 50 cells.mm^−3^ [8], and quantified the expression of MMPs from five different MMP subgroups such as collagenase (MMP 1), gelatinase (MMP 2 and MMP 9), matrilysin (MMP 7), stromelysin (MMP 3, MMP 10, and MMP 11), membrane type (MMP 14), and tissue inhibitors of metalloproteinases (TIMPs) (TIMP1, 2, 3, and 4) (Table 2). We determined that, for both cell lines, the expression of these MMPs was upregulated at high cell density conditions (Figure 1G). mRNA expression of TIMPs in matrix embedded fibrosarcoma cells demonstrates that cell-density significantly increased TIMP3 and TIMP4 expression but did not impact TIMP1 or TIMP2 (Supplementary Figure 1D–1G).

**Table 1:** shRNA sequences for knockdowns

*MMP1 sh372996*	CCGGCTTGAAGCTGCTTACGAATTTCTCGAGAAATTCGTAAGCAGCTTCAAGTTTTTG
*MMP1 sh372993*	CCGGGCTAACCTTTGATGCTATAACCTCGAGGTTATAGCATCAAAGGTTAGCTTTTTG
*MMP7 sh304204*	CCGGTTGCAGAATACTCACTATTTCCTCGAGGAAATAGTGAGTATTCTGCAATTTTTG
*MMP7 sh304140*	CCGGCCACTCCATTTAGCAATTATGCTCGAGCATAATTGCTAAATGGAGTGGTTTTTG
*MMP9 sh373061*	CCGGGCCGGATACAAACTGGTATTCCTCGAGGAATACCAGTTTGTATCCGGCTTTTTG
*MMP9 sh373007*	CCGGGATGCGTGGAGAGTCGAAATCCTCGAGGATTTCGACTCTCCACGCATCTTTTTG
*MMP11 sh50713*	CCGGGATGTCCACTTCGACTATGATCTCGAGATCATAGTCGAAGTGGACATCTTTTTG
*MMP11 sh57016*	CCGGTCCCGAGAAGAACAAGATCTACTCGAGTAGATCTTGTTCTTCTCGGGATTTTTG

Pharmacological intervention of IL-6R and IL-8R using Tocilizumab and Reparixin (T+R) suppresses cell-density-dependent migratory potential in tumorigenic, metastatic cells [8]. Tocilizumab is a humanized monoclonal antibody that targets the receptor of IL-6 and Reparixin is a small molecule that targets the receptor of IL-8. Considering the role that MMPs play in regulating cell migration, and that cell density regulates MMP production through the synergistic signaling of IL-6 and IL-8, we speculated that treatment of cells with T+R would down-regulate MMP production. HT1080 cells embedded in a 3D collagen I matrix were treated with T+R, and then were analyzed for MMPs expression using PCR studies. We observed that the expression of MMP 1, 2, 3, 9, and 10 were greatly decreased when the cells were treated with T+R. The expression of MMP 14 was unaffected by the treatment while, strikingly, the expression of MMP 11 was greatly increased in the treated condition (Figure 1G).

We further tested the effect of T+R on MMP 1 activity and found that it was significantly decreased with the treatment of T+R (Supplementary Figure 1C). In sum, these findings suggest that MMP expression is regulated by cell density through the synergistic paracrine signaling pathway of IL-6 and IL-8 where MMP expression is increased at both an RNA and protein level, resulting in an increased MMP activity.

The janus kinase/signal transducer and activator of transcription (JAK/STAT) pathway relays signals from extracellular polypeptide signals, through transmembrane receptors, directly to target gene promoters in the nucleus to provide a mechanism for transcriptional regulation without secondary messengers [23] JAK/STAT signaling is implicated in the regulation of MMPs production through IL-6 and IL-8 independently. For instance, IL-6 regulates MMP 10 through JAK2/STAT3 signaling in adenocarcinomas [10–13]. Additionally, local tumor cell density regulates cell density-dependent phenotypes through the synergistic signaling of IL-6 and IL-8 via the JAK2/STAT3 pathway [8]. We thus hypothesized that JAK2/STAT3 signaling was involved in the cell density-dependent regulation of MMPs. Indeed, the expression of JAK2 and STAT3 are significantly upregulated in matrix embedded cells at HD (Supplementary Figure 1H and 1I). We further verified this observation by treating matrix embedded fibrosarcoma cells with inhibitors of JAK2 and STAT3. Cells treated with these inhibitors showed an overall decreased expression of MMPs from the different subgroups and TIMPs (Supplementary Figure 1J). This observation, coupled with the finding that MMP expression is upregulated at HD, suggests that local tumor cell density regulates MMP production through the synergistic signaling of IL-6 and IL-8 via the JAK/STAT pathway [24–26] (Figure 1H).

### Cell density-dependent role of MMPs in the regulation of cancer cell migration

**Figure 2:**
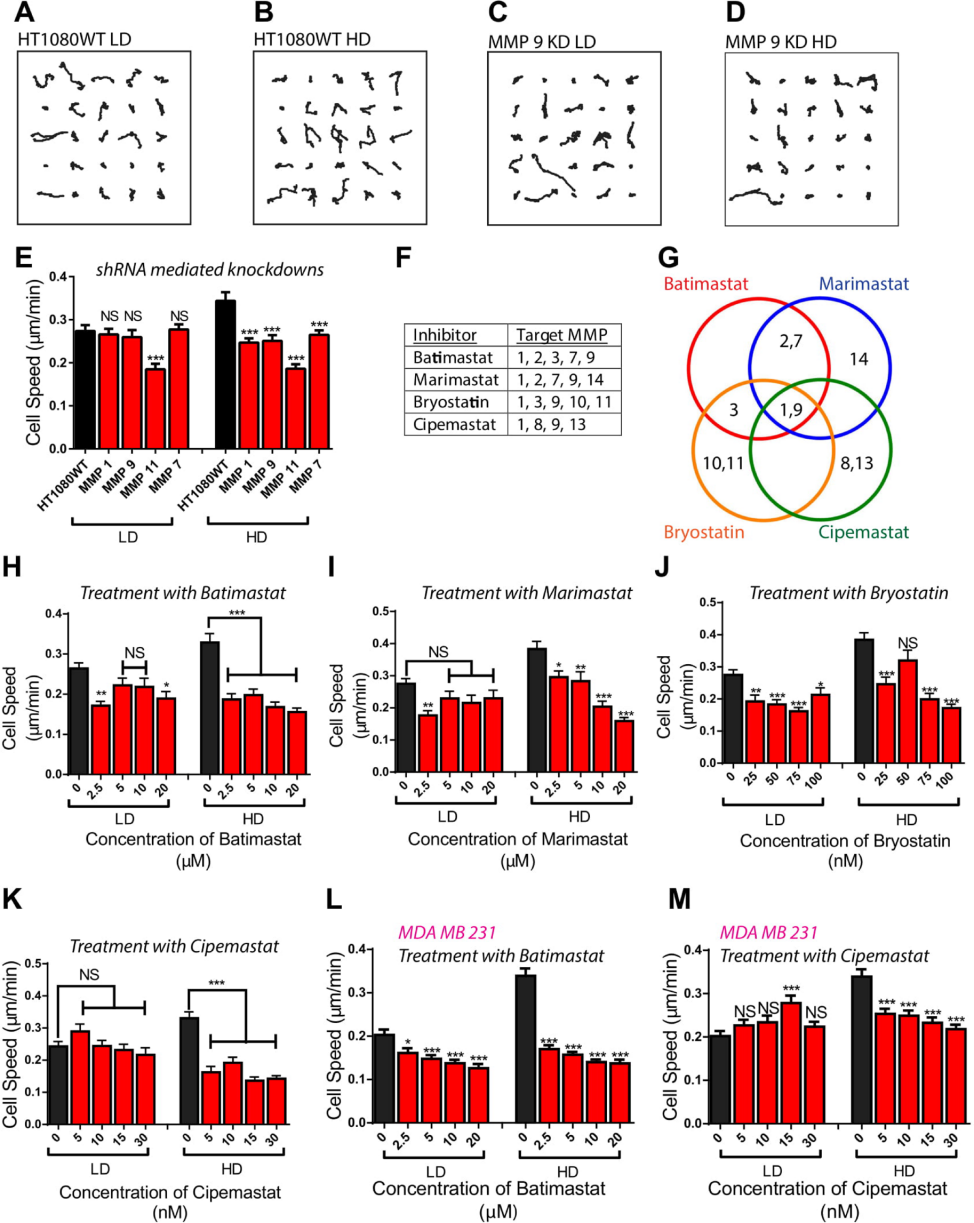
Depletion/inhibition of MMPs induce different migratory phenotypes at low and high cell densities (**A**–**D**) Trajectories of wild type and MMP 9 knockdown HT1080 fibrosarcoma cells at low and high cell densities. (**E**) Depletion of MMPs using shRNA mediated interference demonstrates different migratory phenotypes at low and high cell density. (**F**, **G**) MMPs that specific Matrix Metalloproteinase Inhibitors (MMPIs) target. (**H**–**K**) Treatment of fibrosarcoma cells with MMPIs demonstrates different migratory phenotypes at low and high cell density. (**L**, **M**) Treatment of carcinoma cells with MMPIs demonstrate different migratory phenotypes at low and high cell density. In all panels, data is represented as mean ± s.e.m from three independent experiments. ^*^P < 0.05; ^**^P < 0.01; ^***^P < 0.001(ANOVA) (*n* = 3).

Considering that MMPs may play a critical role in cancer cell migration [27], and that cell density plays an integral role in the production of MMPs, we investigated the effect of knocking down specific MMPs from the different subgroups on cell density-dependent migration (Table 1). In cell density-dependent migration, tumorigenic, metastatic cells at a HD condition migrate significantly faster than those at a LD condition [8]. Cell migration parameters within the matrix at both densities were monitored for 16.5 h using live-cell phase-contrast microscopy at a rate of a 30 frames/h [28–30]. Strikingly different migration patterns were observed at LD and HD for these different cell lines (Figure 2A–2D and Supplementary Figure 2A–2F). Based on previous studies, we would have expected cell migration to significantly decrease in the shRNA-mediated knockdowns at both LD and HD [31, 32]; however, depleting cells of MMP 1, 9, and 7 had no significant effect on cell speed at the LD condition. For the HD condition, cell speed was significantly decreased for cells with shRNA-mediated knockdowns of MMP 1, 9, and 7. Depletion of MMP 11 significantly decreased the migration of cells at both density conditions, indicating that it may play a critical role in migration and may not be regulated by cell density (Figure 2E). This observation is further reinforced by our PCR data that showed that the expression of MMP 11 was greatly increased when cells were treated with T+R (Figure 1G).

**Table 2:** Primer sequences for PCR studies

HS-18S-FWD	GAGGATGAGGTGGAACGTGT
HS-18S-REV	AGAAGTGACGCAGCCCTCTA
MMP 1 FWD	AAAATTACACGCCAGATTTGCC
MMP 1 RVS	GGTGTGACATTACTCCAGAGTTG
MMP2 FWD	TACAGGATCATTGGCTACACACC
MMP2 RVS	GGTCACATCGCTCCAGACT
MMP 3 FWD	CTGGACTCCGACACTCTGGA
MMP3 RVS	CAGGAAAGGTTCTGAAGTGACC
MMP 7 FWD	GAGTGAGCTACAGTGGGAACA
MMP 7 RVS	CTATGACGCGGGAGTTTAACAT
MMP 9 FWD	AGACCTGGGCAGATTCCAAAC
MMP 9 RVS	CGGCAAGTCTTCCGAGTAGT
MMP 10 FWD	TGCTCTGCCTATCCTCTGAGT
MMP 10 RVS	TCACATCCTTTTCGAGGTTGTAG
MMP11 FWD	CCGCAACCGACAGAAGAGG
MMP 11 RVS	ATCGCTCCATACCTTTAGGGC
MMP14 FWD	GGCTACAGCAATATGGCTACC
MMP 14 RVS	GATGGCCGCTGAGAGTGAC
TIMP 1 FWD	TGTTGCTGTGGCTGATAG
TIMP 1 RVS	CTGGTATAAGGTGGTCTGG
TIMP 2 FWD	ACGATATACAGGCACATTATG
TIMP 2 RVS	GGTCAGGAGTCTTAACAGG
TIMP 3 FWD	GGTGAAGCCTCGGTACATCT
TIMP 3 RVS	AGGACGCCTTCTGCAACTC
TIMP 4 FWD	TTTCTTCTGGCTTAGTCTGTTTTCT
TIMP 4 RVS	ATTCGCCATTTCTCCCCTACCA

We also investigated the functional effect of these knockdowns on the persistence time along the primary axis of migration as highlighted by Wu *et al*. [28, 33], and found that the persistence of migration was significantly decreased in both LD and HD conditions. (Supplementary Figure 2G).

### Cell density affects cell response to MMP inhibitors

Based on the above analysis of MMP-depleted cell lines, we hypothesized that different migratory phenotypes should also be observed if cells at LD and HD were treated with MMPIs. To test this hypothesis, we treated matrix embedded HT1080 fibrosarcoma cells at LD and HD with Batimastat, Marimastat, Cipemastat, and Bryostatin-1 [6, 34, 35] (Figure 2F and 2G). Both Batimastat and Marimastat are popular MMPIs that were unsuccessful in clinical trials. Batimastat was declared unsuitable as it could not be administered orally and caused peritonitis [35]. Marimastat did not prolong progression-free survival in patients with metastatic breast cancer [36]. Cipemastat is considered a more efficient MMPI that targets the collagenase subgroup instead of stromelysin, and gelatinase [37]. This MMPI was developed by Roche as a treatment for rheumatoid and osteoarthritis, but was unsuccessful in clinical trials as it did not prevent progression of joint damage in patients with rheumatoid arthritis [38]. Bryostatin-1 is a potent modulator of protein kinase C (PKC) [39], and while it does not directly affect the activity of MMPs, it can inhibit the production of MMP-1, 3, 9, 10 and 11 through the inhibition of PKC [40]. As observed with the MMP knockdown cell lines, different migratory phenotypes were detected at LD and HD conditions when the cells were treated with the MMPIs. Treatment of matrix embedded cells at LD with Batimastat, Marimastat, Cipemastat, and Bryostatin-1 had a slight effect on cell speed; however, the same treatment of matrix-embedded cells at HD did significantly decrease cell speed (Figure 2H–2K and Supplementary Figure 3A–3H). Treatment of LD and HD matrix embedded cells with the four MMPIs decreased persistence significantly (Supplementary Figure 3I–3L). Our findings suggest that Bryostatin-1 may be more effective than Batimastat, Marimastat, and Cipemastat as it consistently decreased cell speed at LD. This result may be because Bryostatin-1 is the only MMPI in our investigation that targeted MMP 11.

Similar migration patterns were observed in matrix-embedded breast carcinoma cells MDA-MB-231 at LD and HD treated with Batimastat and Cipemastat (Figure 2L and 2M, and Supplementary Figure 3M and 3N). These observations suggest that local tumor cell density plays a role in the responsiveness of tumor cells to MMPIs, and could be an underlying fundamental reason for the failure of these MMPIs in clinical trials.

### MMPs are downregulated *in vivo* through a tandem tocilizumab/reparixin therapeutic intervention

**Figure 3:**
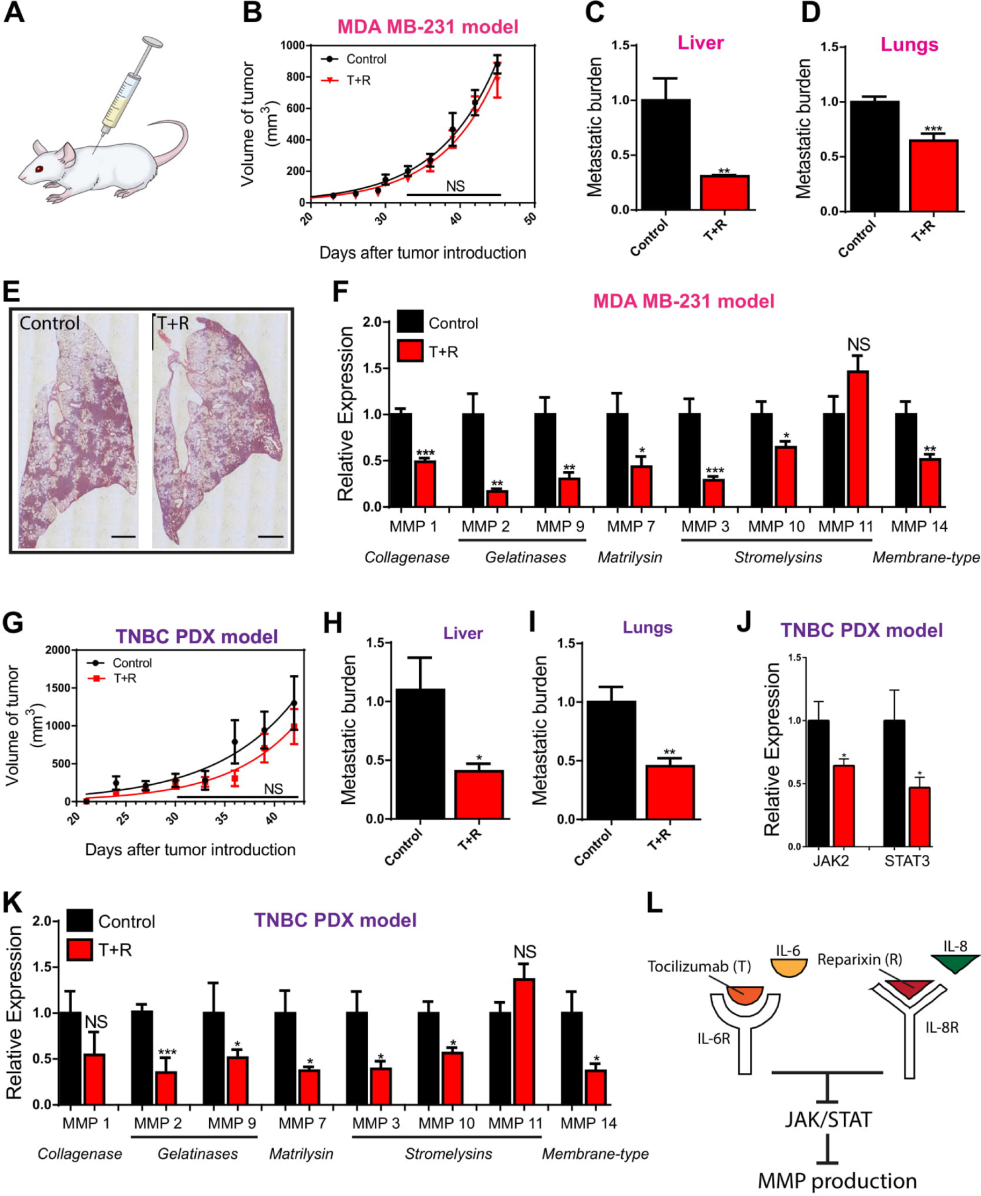
*In vivo* validation (**A**) Illustration depicts mouse being injected. (**B**) Rate of tumor growth is unaffected by treatment with Tocilizumab and Reparixin (T+R) in the MDA-MB-231 mouse xenograft model. (**C**, **D**) Metastatic burden is decreased in mice treated with T+R in the MDA-MB-231 model. (**E**) Lungs excised and stained with hematoxylin and eosin demonstrate fewer metastases in the T+R treated group. (**F**) Expression of MMPs in group injected with T+R relative to the control group injected with saline in the MDA-MB-231. (**G**) Rate of tumor growth is unaffected by treatment with Tocilizumab and Reparixin (T+R) in the patient derived triple negative breast cancer model (TNBC PDX). (**H**, **I**) Metastatic burden is decreased in mice treated with T+R in the TNBC PDX model. (**J**) Decreased expression of JAK2 and STAT3 in the T+R treated mice in the TNBC PDX model. (**K**) Expression of MMPs in group injected with T+R relative to the control group injected with saline in the TNBC PDX model. (**L**) Illustration depicts that pharmacological intervention with Tocilizumab and Reparixin suppresses JAK/STAT signaling which in turn suppresses MMP production. In all panels, data is represented as mean ± s.e.m of five mice. ^*^P < 0.05; ^**^P < 0.01; ^***^P < 0.001(ANOVA)

Considering the role of MMPs in clinical annotations, such as survival and metastasis, and the failure of MMPIs in clinical studies, we sought to develop a new approach of targeting MMPs to improve clinical outcomes for patients. Inhibition of IL-6R and IL-8R using T+R can decrease metastatic burden of cancer cells via the suppression of cell density-dependent migration [8]. We hypothesized that this pharmacological intervention could inhibit the cell density-dependent regulation of MMPs and decrease the expression of MMPs, thus contributing to the decrease in effective metastasis observed (Figure 3A–3E). The effect of these drugs on the expression of MMPs and thus metastasis was examined by generating an animal model through the introduction of MDA-MB-231 breast cancer cells into the mammary fat pad of NSG (NOD SCID Gamma) mice. Mice were injected with either saline or T+R every three days for 6 weeks. As predicted from our *in vitro* results, we observed that the treatment significantly decreased the expression of JAK2, STAT3 (Supplementary Figure 4A), and MMPs in the subgroups of collagenase, gelatinase, matrilysin, and membrane type (Figure 3F). In the stromelysins subgroup, the expression of MMP 3 and 10 was significantly decreased in the treated group. As expected, the treatment had no effect on the expression of MMP 11 (Figure 3F).

We further verified our *in vivo* observations using a subcutaneous patient-derived triple negative breast cancer mouse xenograft model (TNBC PDX model). As with the MDA-MB-231 model, a set of mice was injected with saline as a control, and another set with T+R every three days for 6 weeks. As expected, tumor growth was unaffected by the T+R treatment, while metastatic burden to the liver and the lungs were significantly decreased in the treated group (Figure 3G–3I). Similar to the MDA-MB-231 model, the T+R treatment significantly decreased the expression of JAK2, STAT3, and MMPs in the subgroups of collagenase, gelatinase, matrilysin, and membrane type. MMP 11 was unaffected by the pharmacological intervention of T+R (Figure 3J and 3K). Findings from the two animal models suggest that inhibiting the binding of IL-6 and IL-8 to their cognate receptors can down regulate the production of MMPs through the suppression of the JAK2/STAT3 pathway. (Figure 3L).

Our *in vitro* and *in vivo* findings demonstrate that MMPs are regulated by cell density through the synergistic paracrine signaling of IL-6 and IL-8 via the JAK/STAT pathway. Pharmacological intervention of this pathway using T+R significantly down regulates the production of MMPs and may contribute to the decrease in metastatic burden. Considering that previous clinical trials with MMPIs have failed, our study suggests that T+R can be used to alternatively target MMP activity to reduce metastatic capacity of tumor cells and improve patient outcomes.

### MMP expression in triple-negative breast cancer PDX mouse model

**Figure 4:**
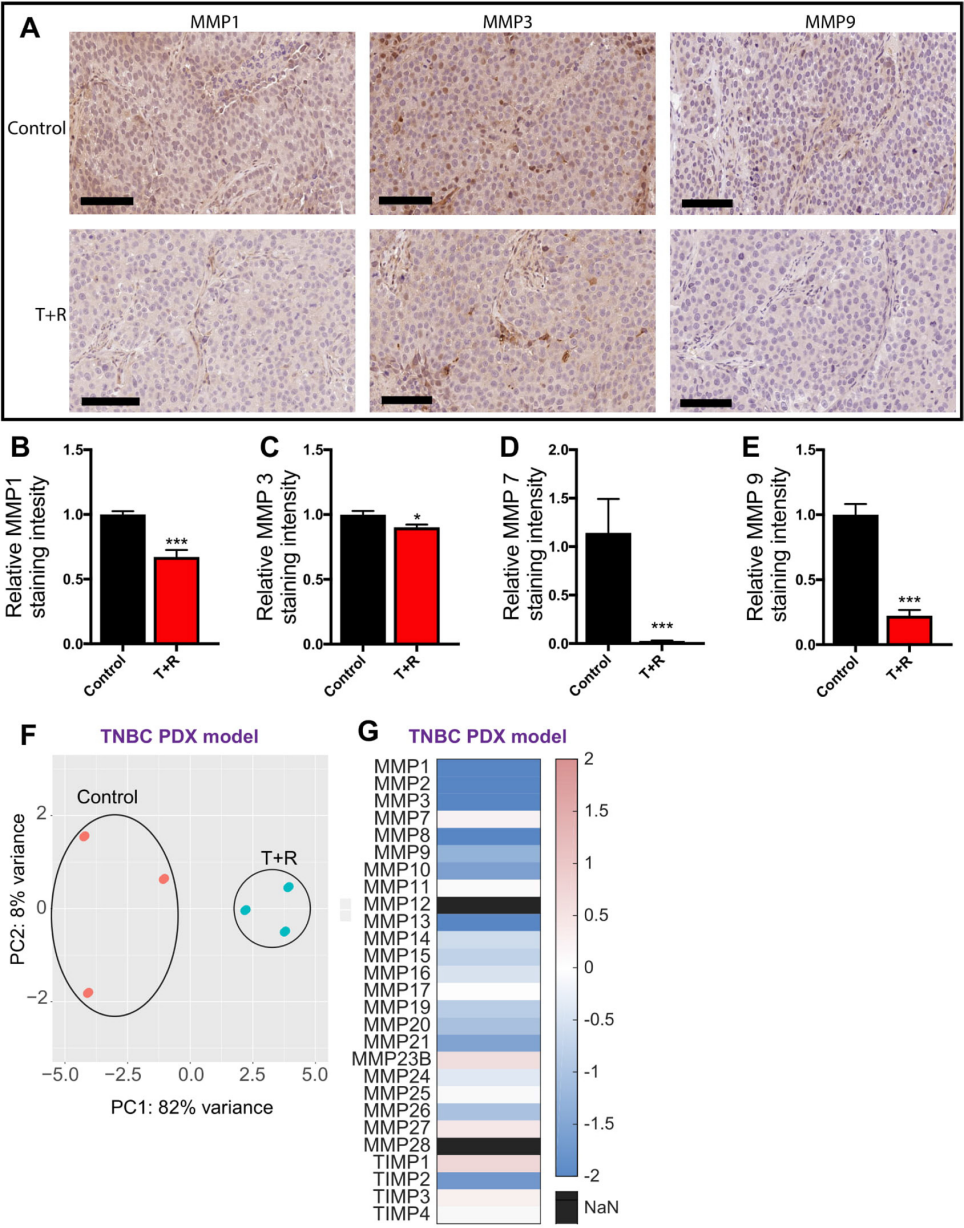
RNA sequencing of PDX tumors (**A**) Representative immunohistochemical staining with MMP1, MMP3, MMP7, and MMP9 demonstrate decreased expression in the tumors obtained from mice treated with T+R. Scale bar, 100 μm. (**B**–**E**) Decreased protein expression of MMP1, MMP3, MMP7, and MMP9 in the PDX tumors obtained from mice treated with T+R (**F**) Principle component analysis (PCA) of the top 1,000 most significant genes (**G** and E) Heat map demonstrating MMPs and TIMPs upregulated/downregulated (Log2FC) following treatment with a combination of tocilizumab and reparixin.

To further verify the downregulation of MMP expression through the inhibition of cell density dependent mechanisms, we conducted immuno-histochemical staining for MMP 1, 3, 7, and 9 and found a markedly decreased expression of the these MMPs in tumors from mice treated with the combination of drugs (Figure 4A–4F).

We also conducted RNA sequencing of the PDX tumors from the pre-clinical mouse models. Principal component analysis (PCA) was performed on the top 1,000 most significant genes to study the relationship of global transcriptomes. The analysis demonstrated that the transcriptomes of control and T+R for each of the three tumors sequenced clustered in close proximity and similar quadrants. The two treatment groups, however, clustered in different quadrants, indicating significant phenotypic differences (Figure 4F).

We also used the data from RNA sequencing to gain further insights into the down regulation of MMPs in the PDX tumors that were treated with T+R. Of the 23 MMPs and 4 TIMPs that we investigated 17 were downregulated in the T+R condition. As shown by our PCR data, expression of MMP 11 was unaffected by the treatment (Figure 4G). This data together with the previous data demonstrates that T+R successfully downregulates MMP expression which contributes to the decreased metastasis observed with the T+R treatment.

## DISCUSSION

The results of this study suggest that the expression of MMPs in tumor cells is regulated by cell density through the synergistic paracrine mechanism of IL-6 and IL-8 via the JAK2/STAT3 signaling pathway. The findings show that the activity of collagenases, gelatinases, stromelysins, and membrane type MMPs are increased at HD. Depletion or inhibition of these subgroups of MMPs has little to no effect on the migratory phenotype of tumor cells in a 3D collagen I matrix at LD. In contrast, depletion or inhibition of these MMP subgroups significantly decreases the speed of matrix-embedded cells at HD. Similar migratory phenotypes and drug responsiveness can be observed when matrix-embedded fibrosarcoma and carcinoma cells are treated with MMPIs such as Batimastat, Marimastat, Cipemastat, and Bryostatin.

These findings indicate that local tumor cell density plays a critical role in the responsiveness of tumor cells to therapeutic agents. MMPIs have little to no effect on cell migration, a key driver of metastasis, at LD conditions, which could account for the failure of MMPIs, particularly Marimastat in phase II clinical trials. This result is in line with previous studies that demonstrated that cells at LD are more effective at invading an endothelial monolayer [41]. This outcome emphasizes the necessity of adopting a more robust testing platform that uses 3D cultures that consider tumor cell density as an important parameter. These 3D cultures with known cell densities will more accurately represent *in vivo* conditions including spatiotemporal gradients of biochemical cues such as cytokines and growth factors [29, 42]. Further, by recognizing the importance of cell density in 3D cell migration studies, more meaningful comparisons across independent studies may be performed from data obtained from different assays such as enzyme-linked immunosorbent assays (ELISAs), Luminex multiplex assays, and PCRs.

Interestingly, our expression and migration results indicate that unlike the majority of MMPs, MMP 11 is not regulated through cell density via the signaling of IL-6 and IL-8. MMP 11 is a unique matrix metalloproteinase that does not share any function with the other MMPs involved in malignant processes. MMP 11 is not able to degrade any major ECM component, it does not modify epithelial cell proliferation or motility, and it does not appear to be a pro-angiogenic or a pro-apoptotic factor. MMP 11 in fact exhibits anti-apoptotic function, a first-known activity for a MMP [43, 44]. Although MMP 11 expression has previously been correlated with an aggressive profile and invasiveness in different cancers [45, 46], our findings suggest that targeting MMP 11 has no effect on suppressing cell density-dependent migration and thus metastasis.

Over 50 MMPIs have already proven to be ineffective in clinical trials. While the reasons for their failures may vary, alternate strategies to target expression of MMPs should be explored. As our findings suggest that MMP expression is regulated through cell density via the synergistic paracrine mechanism of IL-6 and IL-8, we recommend using a cocktail of T+R as an alternative method of targeting MMP production. Our *in vitro* and *in vivo* data demonstrate that MMP expression can be significantly decreased with this combination of therapeutics. Our *in vivo* data from two independent mouse models suggests that treatment with T+R significantly decreases the expression of MMPs, and thus contributes to the decrease of metastatic capacity of cancer cells with minimal systemic toxicity

This study provides new insights into the regulation of MMP expression in tumor cells. MMPs have been implicated in cancer metastasis and have been targeted in multiple clinical trials that have surprisingly failed. Our study demonstrates that MMP expression is regulated by local tumor cell density through the synergistic paracrine signaling of IL-6 and IL-8. Based on our findings, we suggest a new strategy to target MMP expression and to prevent or treat cancer metastasis.

## MATERIALS AND METHODS

### Cell culture

Human fibrosarcoma HT1080 cells (obtained from ATCC) were cultured in Dulbecco's modified Eagle's medium (DMEM, Mediatech) supplemented with 10% (v/v) fetal bovine serum (FBS, Hyclone Laboratories), and 0.005% (w/v) gentamicin (Quality Biological). Human breast carcinoma MDA-MB-231 cells (obtained from ATCC) were cultured in DMEM (Mediatech) supplemented with 10% FBS (Hyclone) and 1% penicillin-streptomycin. The cells were maintained at 37° C and 5% CO_2_ in a humidified incubator during cell culture and live-cell microscopy. All cell lines were tested for mycoplasma and deemed free of contamination.

### Depletion of protein with shRNAs

HT1080 cells were transfected by the same procedure previously outlined in [29]. shRNA constructs targeting the MMP 1, MMP 9, MMP 7, MMP 11 genes were purchased from Sigma Aldrich. PCR studies were performed after lentiviral mediated transduction and only shRNAs showing more than 85% knockdown were used for subsequent studies (Supplementary Figure 2H–2K). Genomic sequences for the knockdowns are listed in Table 1.

### 3D collagen I matrix

HT1080 cells were embedded in 2 mg/ml type I collagen gel as described previously by Fraley *et al.* [30]. Briefly: cell suspensions containing 5,000 (low cell density environments) and 25,000 (high cell density environments) cells in 1:1 (v/v) ratio of cell culture medium and reconstitution buffer were mixed with the appropriate volume of soluble rat-tail collagen I (Corning Inc.) to obtain a final collagen I concentration of 2 mg/ml. A calculated amount of 1 M NaOH was quickly added, and the final solution was mixed well to bring the pH to ~7. The cell suspension was added to a 24-well coverslip-bottom cell-culture dish, and immediately transferred to an incubator maintained at 37° C to allow polymerization of the collagen. Fresh medium and medium containing specific concentrations of MMP inhibitors were added 1 h before imaging. MDA-MB-231 cells were embedded in 1 mg/ml type I collagen matrix with 5,000 cells/well to simulate low cell density environments, and 50,000 cells/well to simulate high cell density environments.

### Speed and protrusion topology of matrix embedded cells

Phase-contrast microscopy was conducted on matrix-embedded cells. Images were collected at 2 min intervals for 16.5 h using a Cascade 1K CCD camera (Roper Scientific) mounted on a Nikon TE2000 microscope with a 10× objective lens to obtain 500 frames. The frames were converted into movies, from which a minimum of 50 single cells were tracked using Metamorph imaging software for each condition. A custom MATLAB program calculated the velocity for each cell using the *x*- and *y*-coordinates obtained from the tracking data using the following equation:

$$\text{Speed}=\frac{\sqrt{<[x(t+\Delta t)-x(t)]2+[y(t+\Delta t]-y(t)]2>.}}{t}$$

Equation 1: Cell speed calculation

Mitotic cells were not included in the measurements. Persistence time over primary axis (Pp) was obtained by the methods described by Wu *et al.* [28, 33].

### Luminex assay

HT1080WT cells were embedded in type 1 3D collagen matrices in increasing cell numbers from 5,000 to 75,000 per well. Collagen gels were prepared to obtain a final collagen concentration of 2 mg ml^−1^ using soluble rat-tail collagen I (Corning). Cell supernatants were extracted 24 hours after incubation at 37° C in a humidified incubator.

A Luminex High Performance Assay multiplex kit was obtained from R&D systems (Biotechne). All reagents were prepared following the instructions in the kit, specifically for cell supernatants. The assay plate was read using a Magpix Multiplexing system (Luminex). Data was analyzed using xPONENT software to determine secreted protein content (Luminex).

### PCR methods

Expression of MMP 1, 2, 3, 7, 9, 10, 11, and 14 were measured using qRT-PCR for the cell densities of 10 cells.mm^−3^ and 50 cells.mm^−3^. Complete RNA extraction was performed with RNA MiniPrep kit (Zymo research). cDNA synthesis was carried out as previously described by Gilkes *et al.* [47]. The sequence for the cDNA primers that were used during PCR are listed in Table 2.

### Inhibitor assays

Matrix embedded cells with low and high cell densities were exposed to MMP inhibitors: Batimastat (Sigma Aldrich, CAS: 130370-60-4), Bryostatin-1(Sigma Aldrich, CAS: 83314-01-6), Marimastat (Sigma Aldrich, CAS: 154039-60-8), Cipmestat (Santa Cruz Biotechnology, CAS 190648-49-8) for 1 h before cells were imaged as described under *Speed and protrusion topology of matrix embedded cells* section above.

### *In vivo* mouse modeling

Studies using NSG (NOD SCID Gamma) mice were carried out according to protocols approved by the Johns Hopkins University Animal Care and Use Committee in accordance with the NIH Guide for the Care and Use of Laboratory Animals. All mice were housed at a temperature of 25° C under a 12-hr dark/light cycle. Tocilizumab and Saline for injection were obtained from the research pharmacy of The Johns Hopkins Hospital. Reparixin was obtained from Cayman Chemical and Med Chem Express.

### Pre-clinical MDA-MB-231 breast cancer model

NSG mice for this study were obtained from Johns Hopkins Medical Institution. Human breast cancer, MDA-MB-231, cells harvested by trypsinization were resuspended at 10^7^ cells/mL in a 1:1 mix of PBS:Matrigel and 1 × 10^6^ cells were injected into the mammary fat pad (MFP) of 5–7 week-old mice. 10 days after the cell injections, the five mice received a subcutaneous injection of Tocilizumab (25 mg/kg) and Reparixin (30 mg/kg). Another five mice received 100 μL of a saline solution as a control. Primary tumors were measured in two dimensions (a and b), and volume was calculated as 4/3π ((a × b)/2). Mice were sacrificed after 8 weeks.

### Pre-clinical TNBC PDX model

NSG mice with subcutaneous implantations of a triple negative breast cancer cell line, TM00098, were obtained from the Jackson Laboratory. 12 days after the implantation, five mice received an intraperitoneal injection of Tocilizumab (30 mg/kg) and Reparixin (30 mg/kg). Another five mice received 100 μL of a saline solution as a control. Primary tumors were measured in two dimensions (a and b), and volume was calculated as 4/3π ((a × b)/2). Mice were sacrificed after 8 weeks.

For both models, the tumors, livers, and lungs were harvested. Tumors were weighed and processed for RNA isolation and tissue lysate preparation. Genomic DNA was isolated from the organs as per the procedure outlined previously [8]. Expression of MMP 1, 2, 3, 7, 9, 10, 11, and 14 was quantified using PCR methods.

### RNA sequencing

#### RNA isolation

RNA was isolated using Qiagen AllPrep DNA/RNA Mini Kit (catalog # 80204). Agilent BioAnalyzer is used for quality control of the RNA prior to library creation, with a minimum RIN of 8.5.

#### RNA-seq analysis

Sequences were independently aligned to human (GRCh37/hg19) and mouse (GRCm38/mm10) reference genomes using Bowtie2 [5] (version 2.2.8), piped through samtools [6] (version: 1.5) and stored in indexed BAM format. Reads were filtered to include only those which had a mapping quality score greater than or equal to 1. Reads mapped to both human and mouse reference genomes were excluded to restrict to reads that were uniquely mapped to the human genome. DEseq2 R package [7] (version 1.16.1) was used to model read count statistics from replicates across treated vs. untreated samples to identify differentially expressed genes.

### STAT3 and JAK2 inhibitor assays

Collagen I gels were seeded at high cell-density condition and treated with: fresh medium, STAT3 inhibitor (S3I-201, Sigma), or JAK2 inhibitor (AG490, Sigma). After 24 hr incubation, cells were extracted from the matrix and mRNA was isolated from cells using RNA MiniPrep kit (Zymo research). cDNA was prepared as previously described by Gilkes [47]. cDNA kit was purchased from BioRad and prepared for a final concentration of 1000 ng/μl. [47].

### MMP1 and MMP9 activity assay

Collagen I gels were seeded at varying cell densities (utilizing HT1080 cell line) and given fresh medium or 20 μg/μl of tocilizumab and reparixin. Cell supernatant was harvested after 24 hr incubation. MMP activity assays for MMP-1 and MMP-9 were conducted using kits obtained from R&D systems (Biotechne) for cell supernatant. Experiments and analysis were conducted following the provided instructions.

### Immunohistochemistry

Immunolabeling for MMP1, MMP3, MMP7, and MMP9 was performed on formalin-fixed, paraffin embedded sections. Briefly, following dewaxing and rehydration, slides were immersed in 1% Tween-20, then heat induced antigen retrieval was performed in a steamer using Target Retrieval Solution (catalog# S170084-2, Dako) for 45 minutes. Slides were rinsed in PBST and endogenous peroxidase and phosphatase was blocked (catalog# S2003, Dako) and sections were then incubated with primary antibody; anti MMP1 (1:100 dilution; Proteintech# 10371-2-AP,), anti- MMP3 (1:100 dilution; Proteintech; catalog # 17873-1-AP), anti-MMP7 (1:100 dilution; Abcam; catalog# ab4044) and anti- MMP 9 (1:100 dilution; Abcam; catalog # ab38898) for 45 min at room temperature. The primary antibodies were detected by 30 minute incubation with HRP-labeled anti-rabbit secondary antibody (catalog# PV6119, Leica Microsystems) followed by detection with 3,3′Diaminobenzidine (catalog# D4293, Sigma Aldrich,), counterstaining with Mayer's hematoxylin, rehydration and mounting. Intensity of staining was quantified using Aperio ImageScope.

### Statistical analysis

The mean values ± s.e.m were calculated and plotted using GraphPad Prism software (GraphPad Software). One-way ANOVA test was performed to determine statistical significance, which is indicated in the graphs using a Michelin grade scale ^***^p < 0.001, ^**^p < 0.01, and ^*^p < 0.05.

We thank Aimee Dai for her help with illustrations for the manuscript. We also thank Alexandra R. Sneider for her assistance with experiments.

## SUPPLEMENTARY MATERIALS FIGURES AND TABLES


