# Supplementary material for: Tumor cell density regulates matrix metalloproteinases for enhanced migration
Source: Oncotarget. 2018 Aug 24;9(66):32556–69. doi: 10.18632/oncotarget.25863 (PMC6135685; doi:10.18632/oncotarget.25863)
Supplement: Supplementary file 1 [file oncotarget-09-32556-s001.pdf]

Tumor cell density regulates matrix metalloproteinases for enhanced migration

SUPPLEMENTARY MATERIALS

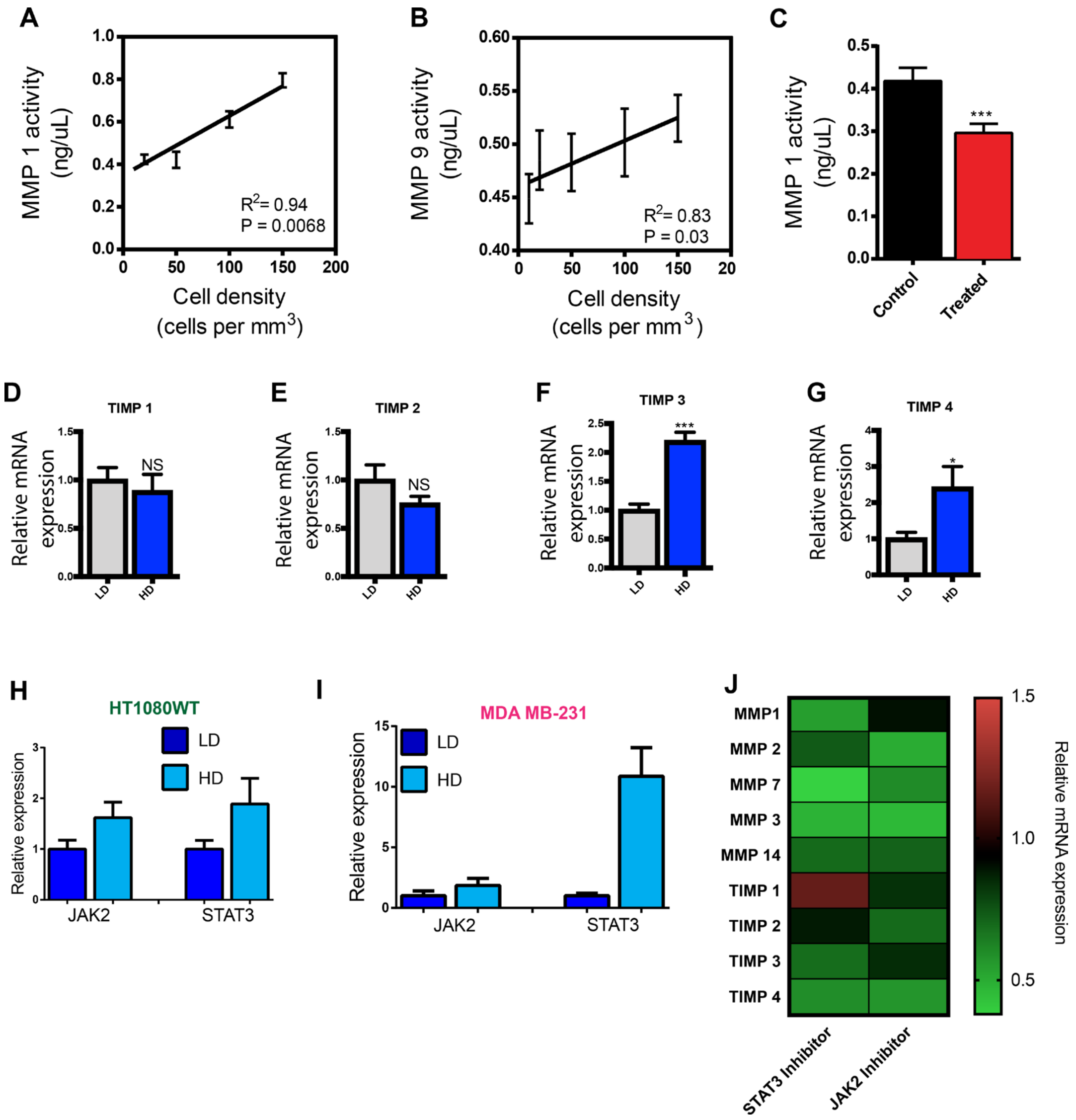

Supplementary Figure 1: (**A** and **B**) Increasing activity of MMP 1 and MMP 9 as a function of cell density (**C**) Activity of MMP 1 is down regulated in in matrix embedded HT1080 fibrosarcoma cells when treated with a combination of anti-IL-6 inhibitor, Tocilizumab, and anti-IL-8 inhibitor, Reparixin. (**D**–**G**) mRNA expression of TIMPs in matrix embedded fibrosarcoma cells demonstrates that cell-density significantly increases TIMP3 and TIMP4 expression but does not impact TIMP1 and TIMP2 (**H** and **I**) Expression of JAK2 and STAT3 is upregulated at a high cell density condition for fibrosarcoma and carcinoma cells. (**J**) Cells treated with inhibitors of JAK2 (AG490) and STAT3 (S3I-201) showed an overall decreased expression of MMPs from the different subgroups and TIMPs.

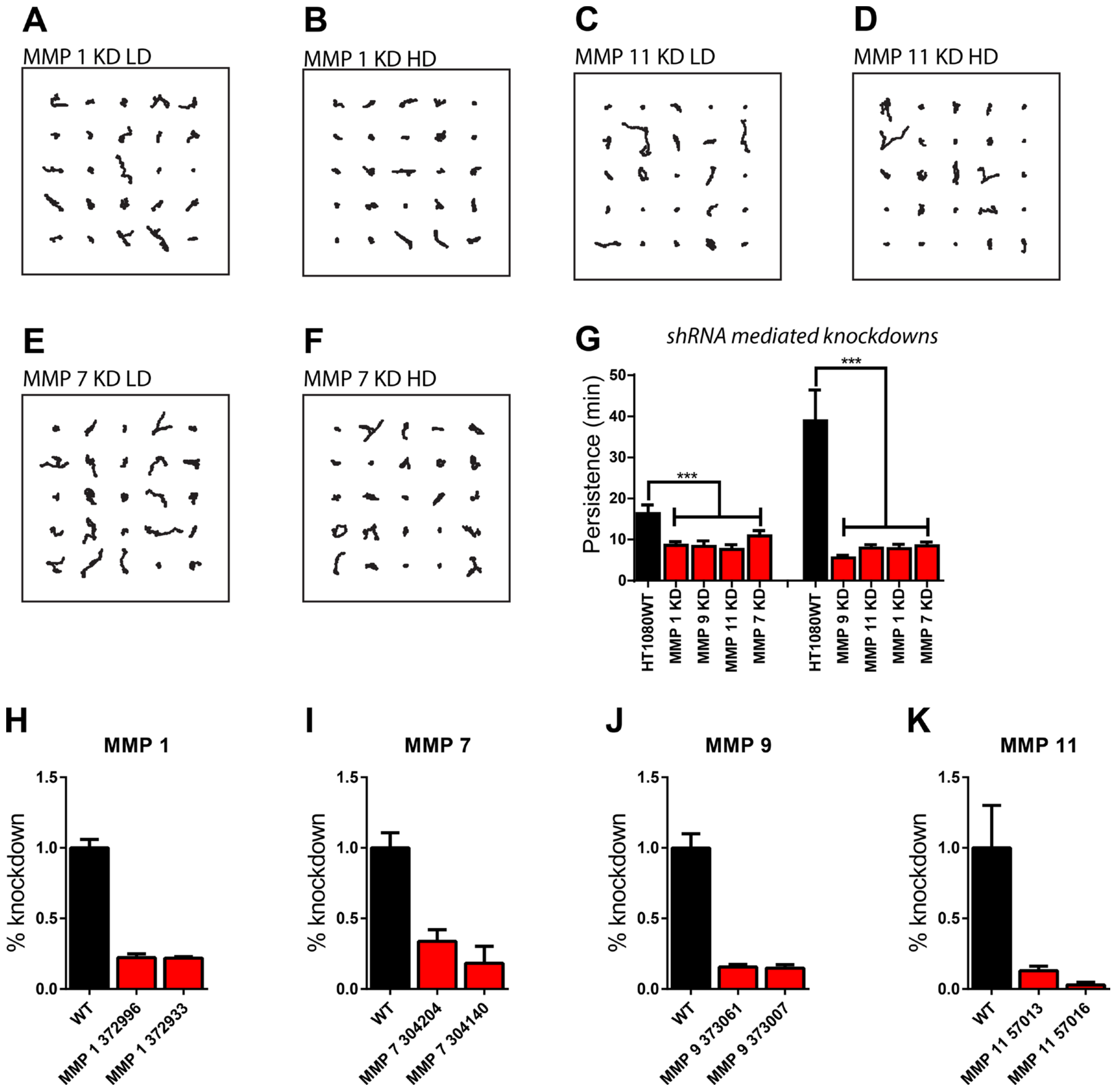

Supplementary Figure 2: (**A**–**F**) Trajectories of MMP 1, MMP 11, and MMP 7 knockdown HT1080 fibrosarcoma cells at low and high cell densities. (**G**) Depletion of MMPs using shRNA mediated interference decreases persistence at both low and high cell density. (**H–K**) Percent depletion of MMP 1, MMP 7, MMP 9, and MMP11 in knockdown cell lines measured at the mRNA level.

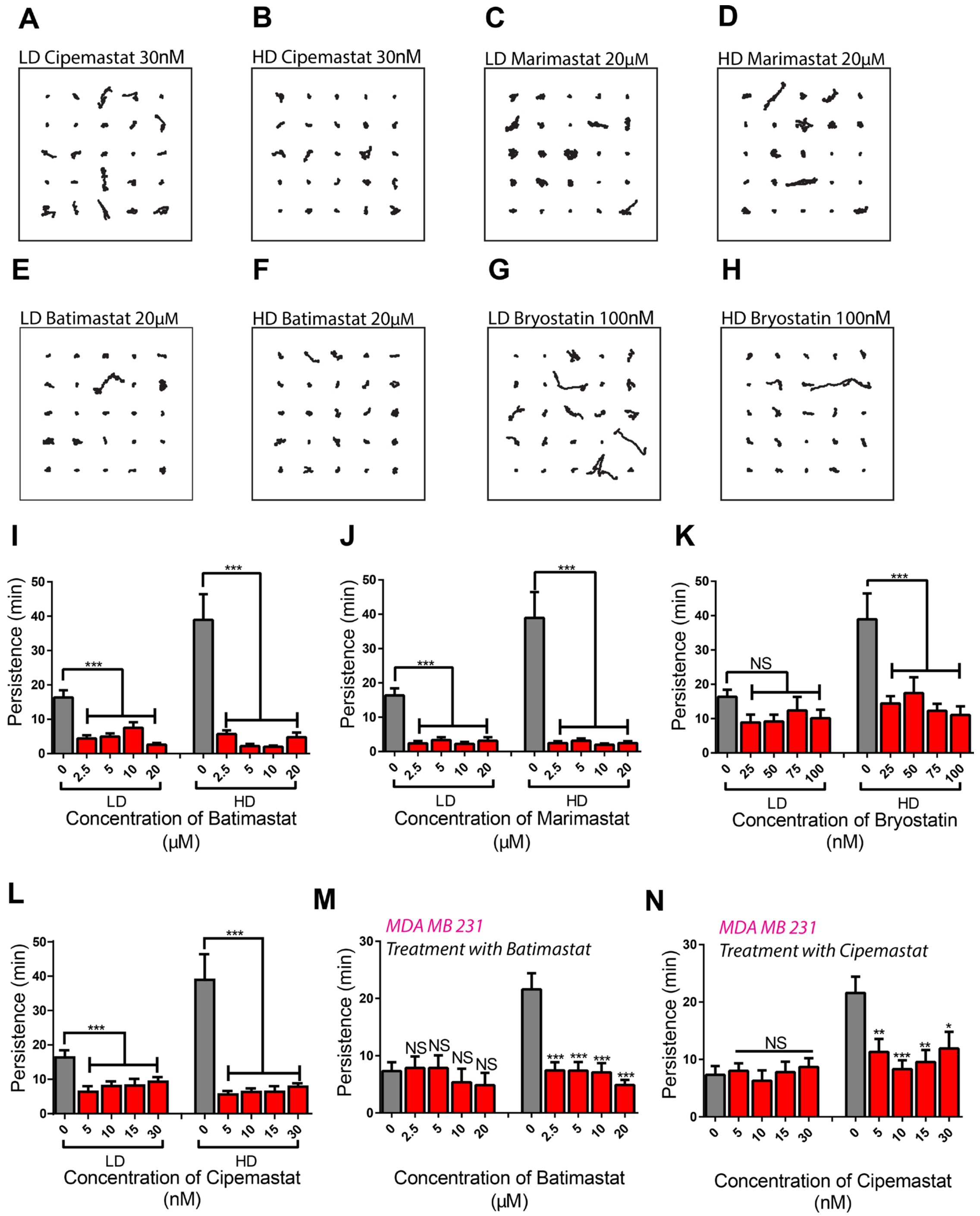

Supplementary Figure 3: (**A**–**H**) Trajectories of HT1080 fibrosarcoma cells treated with Cipemastat, Marimastat, Batimastat, and Bryostatin at low and high cell densities. (**I**–**L**) Treatment of HT1080 fibrosarcoma cells with Cipemastat, Marimastat, Batimastat, and Bryostatin decreased persistence at both low and high cell densities. (**M** and **N**) Treatment of MDA MB 231 breast carcinoma cells with Batimastat and Cipemastat decreased persistence at both low and high cell densities.

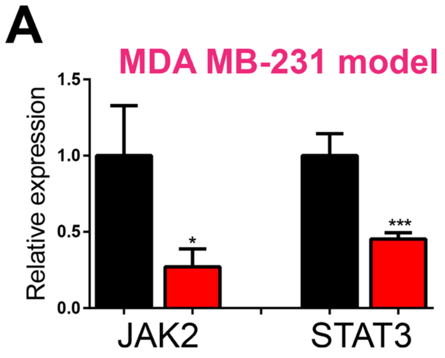

Supplementary Figure 4: (A) Expression of JAK2 and STAT3 is down regulated when animals are treated with Tocilizumab and Reparixin.
